#  Laparoscopic cholecystectomy versus percutaneous catheter drainage for acute calculous cholecystitis in patients over 90 years of age

**DOI:** 10.1007/s00423-023-02903-7

**Published:** 2023-05-13

**Authors:** Camilo Ramírez-Giraldo, Andrés Isaza-Restrepo, Enid Ximena Rico-Rivera, Juan Carlos Vallejo-Soto, Isabella Van-Londoño

**Affiliations:** 1 Hospital Universitario Mayor – Méderi, Bogotá, Colombia; 2 Universidad del Rosario, Bogotá, Colombia

**Keywords:** Elderly, cholecystostomy, cholecystectomy, complications

## Abstract

**Background:**

Laparoscopic cholecystectomy (LC) is the standard of care for acute calculous cholecystitis; however, in patients at high risk for surgery, particularly in the elderly, insertion of a percutaneous catheter drainage (PCD) at gallbladder is recommended. Current evidence suggests that PCD may have less favorable outcomes than LC, but also that LC-associated complications increase in direct relation to patient age. There is no recommendation supported by robust evidence to decide between one or the other procedure in super elderly patients.

**Methods:**

A retrospective observational cohort study was designed to analyze the surgical outcomes of super elderly patients with cholecystitis who underwent LC versus PCD for treatment. The surgical outcomes of a subgroup of high-risk patients were also analyzed.

**Results:**

A total of 96 patients who met the inclusion criteria between 2014 and 2021 were included. The median age of patients were 92 years (IQR: 4.00) with a female predominance (58.33%). The overall morbidity rate in the series was 36.45% and mortality rate was 7.29%. There was no statistically significant difference when compared to the associated morbidity and mortality among patients who underwent LC versus those who underwent PCD, neither in the analysis of the complete series or in the subgroup of high-risk patients.

**Conclusions:**

The morbidity and mortality associated with the two most frequently recommended therapeutic options for operating super elderly patients with acute cholecystitis are high. We found no evidence of superiority in outcomes for either of the two procedures in this age group.

## Introduction

The prevalence of gallstones is estimated to be 10-15% in the general population, its most frequent clinical manifestation being acute cholecystitis. Laparoscopic cholecystectomy (LC) is the current standard of care for acute calculous cholecystitis [1]. However, major complications of LC and postoperative morbidity and mortality rates increase with age, and it is estimated that perioperative mortality risk in those over 80 years of age can be increased tenfold [2]. Additionally, due to their low functional reserve nonagenarian patients are often not considered candidates for general anesthesia, which is necessary for the performance of LC [3]. Therefore, it is essential to explore therapeutic alternatives such as the insertion of a percutaneous catheter drainage (PCD) in the gallbladder for this particular population [1].

There are few studies that assess results of LC in super elderly patients; an age group who, due to recent research, is in a state of constant exponential growth [4, 5]. Moreover, some studies have evaluated PCD management in high-risk patients, finding a higher rate of complications. A systematic review of present literature shows that 30-day mortality after the procedure can be as high as 15.4% [6, 7]. Therefore, it is relevant to compare the results of both management options (LC vs PCD) in patients such as the super elderly, a special population with a high perioperative risk due to their fragility and multiple comorbidities [3]. Such a study has not been described in actual literature up to this date.

The aim of this study is to compare surgical outcomes, complications, and mortality rates in super elderly (> 90 years of age) patients who underwent LC versus PCD for acute calculous cholecystitis.

## Patients and methods

### Study design

A retrospective observational cohort study was designed. Between January 2014 and December 2021, 13.192 cholecystectomies were performed in our institution [8]. Among them, 65 laparoscopic cholecystectomies were carried out in patients over 90 years of age with the presence of cholecystitis. During the same period, 286 PCDs were performed, out of which 31 were performed in patients over 90 years of age coursing with cholecystitis. Variables were collected in an anonymous database. This study was reviewed and approved by the ethics committee (number DVO005 2066-CV1595). We followed STROBE guidelines in order to report this study [9].

### Patients

Patients over 90 years of age with a diagnosis of cholecystitis were included. Patients scheduled for open cholecystectomy with a diagnosis of gallbladder cancer or cholecystectomy associated to another surgical procedure (gastrectomy or pancreaticoduodenectomy, among others), patients without postoperative follow-up (postoperative follow-up appointment), and patients whose records did not have variables of interest were excluded.

In all cases LC indication was a cholecystitis diagnosis confirmed by at least one imaging study, these were classified by severity and were treated accordingly following Tokyo guidelines [10, 11]. The American Society for Gastrointestinal Endoscopy protocol guideline was followed to establish risk of choledocholithiasis; LCs or PCDs was performed without additional studies in low-risk cases; LC or PCD was performed after discarding choledocholithiasis with magnetic resonance cholangiography in intermediate-risk cases and after endoscopic retrograde cholangiopancreatography (ERCP) in high-risk cases [12]. The decision to perform LC or PCD was taken depending on surgeon criteria, taking into account pre-anesthetic assessment and the risk/benefit ratio for the procedure.

All patients had an outpatient control appointment (20 +/- 5 days after hospital discharge) where clinical evolution and surgical wounds were evaluated. In cases with PCD, correct catheter functioning was checked.

We analyzed the following data: patient demographic characteristics, body mass index, ASA Physical Status Classification, presence of comorbidities, preoperative laboratory tests, bile duct diameter in preoperative imaging, classification of cholecystitis severity, need for preoperative ERCP, time from admission to completion of surgical procedure, intraoperative findings (Nassar Score), conversion rate, type of cholecystectomy (total or subtotal), drain usage, PCD removal/dysfunction, complications associated with the procedure and hospitalization, recurrence of biliary disease, hospital stay, reintervention requirement, and mortality.

### Surgical procedure

Laparoscopic cholecystectomy was performed with the standard 4-port technique in the American position. Dissection of the hepatocystic triangle was performed when critical safety window was reached, dissecting above the R4U line in all cases. After reaching the critical view of safety, ligation of the cystic duct and artery and dissection of the gallbladder was performed. In cases where critical view of safety was not achieved, alternative strategies such as fundus first, subtotal cholecystectomy or conversion to open procedure were used according to surgeon preference. Drain placement in surgical site was also performed according to surgeon discretion.

Percutaneous drainage by catheter was performed under ultrasound guidance with a multi frequency convex transducer. The gallbladder was punctured with a Chiba 22 G needle; a micro guide was advanced and a triaxial system was placed over it. Consequently, the micro guide was removed, the support guide was advanced, and the 10 Fr drainage catheter was pushed forward and into the gallbladder. In all cases, the catheter was fixed with a Revolution™ fixation device and plugged into a collection bag for proper drainage.

### Statistical analysis

A description was made with demographic, clinical, paraclinical and surgical variables and outcomes. Categorical variables were described as proportions and continuous variables as medians with their respective interquartile range (IQR). A bivariate analysis was performed with the Chi-squared test for categorical variables and with the Mann-Whitney test for continuous variables among patients who underwent laparoscopic cholecystectomy and those who underwent cholecystostomy. In addition, this same analysis was performed separately on a subgroup of patients defined as high risk (ASA ≥3) in order to compare outcomes. A multivariate analysis was performed to identify the independent factors for major complications (Clavien-Dindo ≥3), the variables were included with *p* < 0.1 in the bivariate analysis and those considered as clinically relevant. This entire analysis was performed in SPSS® 27, considering *p*<0.05 as statistically significant.

## Results

**Fig. 1:**
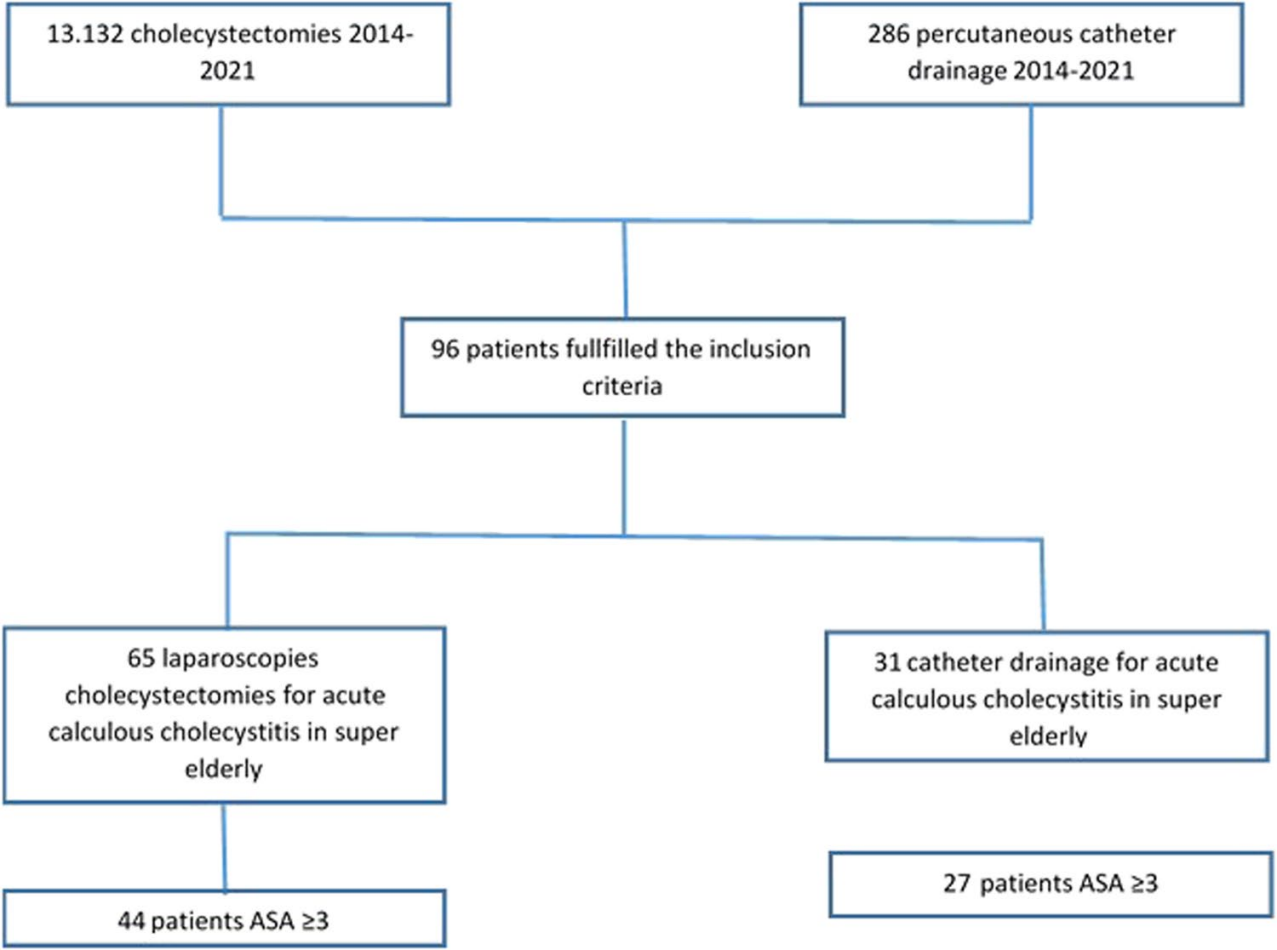
Flowchart of the study selection process

This study included 96 patients. LC was performed in 65 (67.7%) cases. The selection process is shown in the following flowchart (Fig. 1).

Median patient age was 92 years (IQR: 4.00) and were predominantly female (58.33%). Table 1 shows demographic, clinical and paraclinical characteristics according to the procedure performed.

**Table 1:** Demographic, clinical, and surgical characteristics of patients older than 90 years with cholecystitis, according to the procedure performed

	*N* (%) *N***=**96	Laparoscopic cholecystectomy (%) *n***=**65	Percutaneous catheter drainage (%) *n***=**31	*p* value
Age (median)(IQR)(years)	92 (4.00)	92 (3.00)	92 (4.00)	0.214*
Sex				**0.009**
Female	56 (58.33)	32 (49.23)	24 (77.42)	
Male	40 (41.67)	33 (50.77)	7 (22.58)	
Body mass index (median)(IQR)(kg/m^2^)	23.20 (4.65)	23.40 (4.96)	21.95 (3.80)	0.049*
ASA classification				**0.038**
1	0 (0.0)	0 (0.00)	0 (0.00)	
2	25 (26.04)	21 (32.31)	4 (12.9)	
3	65 (67.71)	42 (64.62)	23 (74.19)	
4-5	6 (6.25)	2 (3.08)	4 (12.90)	
Co-morbidity				
Diabetes mellitus	22 (22.92)	16 (24.62)	6 (19.35)	0.566
Arterial hypertension	71 (73.96)	49 (75.38)	22 (70.97)	0.645
Chronic obstructive pulmonary disease	39 (40.62)	22 (33.85)	17 (54.84)	**0.050**
Chronic kidney disease	14 (14.58)	8 (12.31)	6 (19.35)	0.360
Cardiovascular disease	33 (34.38)	13 (20.00)	20 (64.52)	**<0.001**
Liver disease	4 (4.17)	3 (4.62)	1 (3.23)	0.750
Charlson comorbidity index (median)(IQR)(points)	6.00 (3.00)	6.00 (1.50)	7.00 (3.00)	0.773*
Anticoagulants agents	9 (9.38)	3 (4.62)	6 (19.35)	**0.021**
Antiplatelet agents	28 (29.17)	12 (18.46)	16 (51.61)	**<0.001**
Pre-operative laboratories (median)(IQR)				
Leukocytes (x10^3^)	13.15 (6.75)	12.70 (6.55)	14.20 (9.14)	0.087*
Hemoglobin (mg/dL)	13.35 (2.75)	13.70 (2.70)	12.60 (2.70)	0.115*
Bilirubins (mg/dL)	1.17 (1.50)	1.20 (1.63)	1.12 (1.48)	0.826*
Alkaline phosphatase (mg/dL)	127 (128.75)	139 (140.50)	112 (94.00)	0.549*
Aspartate aminotransferase (mg/dL)	30 (83.5)	31 (100.00)	26 (56.00)	0.503*
Alanine aminotransferase (mg/dL)	36.5 (90.75)	40 (123.00)	34 (50.00)	0.146*
Bile duct diameter (median)(IQR)(mm)	10.7 (8.00)	12.7 (6.55)	6 (3.00)	<0.001*
Classification of severity of cholecystitis				**0.022**
I	5 (5.20)	5 (7.69)	0 (0.00)	
II	25 (26.04)	21 (32.31)	4 (12.90)	
III	66 (68.75)	39 (60.00)	27 (87.10)	
Pre-operative ERCP				0.242
No	77 (80.21)	50 (76.92)	27 (87.10)	
Yes	19 (19.79)	15 (23.08)	4 (12.90)	
Time from admission to surgical procedure (median)(IQR) (days)	5.00 (4.75)	5.0 (5.0)	4.0 (5.0)	0.774*
Intraoperative findings (Nassar Score)				
1	11 (11.46)	11 (16.92)	Not apply	
2	10 (10.42)	10 (15.38)		
3	11 (11.46)	11 (16.92)		
4	14 (14.58)	14 (21.53)		
5	19 (19.79)	19 (29.23)		

The *p* values were obtained from the Chi-squared test

*The *p* values were obtained from the Mann–Whitney test

Bold values indicate statistically significant *p* values (*p* < 0.05)

Intraoperative findings were measured using the Nassar score (where a higher score is directly proportional to a more difficult laparoscopic cholecystectomy). We found a higher rate of difficult cholecystectomies (grades 3, 4 and 5) than easy cholecystectomies (grades 1 and 2) [13, 14].

The rate of complications in the complete series was 36.45% and mortality was 7.29%. There were no statistically significant differences when comparing morbidity and mortality among patients who underwent LC versus those who underwent PCD (Table 2).

**Table 2:** Surgical outcomes in patients according to the procedure performed

	*N* (%) *N***=**96	Laparoscopic cholecystectomy (%) *n***=**65	Percutaneous catheter drainage (%) *n***=**31	*p* value
Conversion from laparoscopic to open				
No	56 (58.33)	56 (86.15)	Not apply	
Yes	9 (9.38)	9 (13.85)		
Type of cholecystectomy				
Total	51 (53.12)	51 (78.46)	Not apply	
Subtotal	14 (14.58)	14 (21.54)		
Drain use				
No	44 (45.83)	44 (67.69)	Not apply	
Yes	21 (21.88)	21 (32.31)		
Hospital stay (median)(IQR)(days)	10.50 (6.75)	11.00 (6.50)	10.00 (6.00)	0.677*
Complications				
Bile duct injury	5 (5.21)	5 (7.69)	Not apply	
Bleeding	4 (4.17)	4 (6.15)	0 (0.00)	0.158
Intestinal injury	0 (0.00)	0 (0.00)	0 (0.00)	
Surgical site infection	9 (9.38)	8 (12.31)	1 (3.23)	0.153
Acute myocardial infarction perioperative	1 (1.04)	1 (1.54)	0 (0.00)	0.488
Pulmonary embolism perioperative	2 (2.08)	2 (3.08)	0 (0.00)	0.324
Deep venous thrombosis perioperative	0 (0.00)	0 (0.00)	0 (0.00)	
Health care-associated pneumonia	1 (1.04)	1 (1.54)	0 (0.00)	0.488
Health care-associated urinary tract infection	4 (4.17)	3 (4.62)	1(3.23)	0.750
Pleural efussion	6 (6.25)	5 (7.69)	1 (3.23)	0.398
Removal/dysfunctional drain				
No	28 (29.17)	Not apply	28 (90.32)	
Yes	3 (3.12)		3 (9.68)	
Need for reintervention				0.211
No	88 (91.66)	58 (89.23)	30 (100.00)	
Yes	8 (8.34)	7 (10.77)	1 (3.23)	
Recurrent gallstone related disease	1 (1.04)	0 (0.00)	1 (3.23)	0.145
Clavien-Dindo				0.469
I	10 (10.41)	8 (12.31)	2 (6.45)	
II	7 (7.29)	5 (7.69)	2 (6.45)	
IIIA	4 (4.17)	3 (4.62)	1 (3.23)	
IIIB	4 (4.17)	4 (6.15)	0 (0.00)	
IV	3 (3.12)	3 (4.62)	0 (0.00)	
V	7 (7.29)	5 (7.69)	2 (6.45)	
Any complication	35 (36.45)	28 (43.07)	7 (22.58)	0.051
Major complications (Clavien-Dindo ≥3)	15(15.62)	12 (18.46)	3 (9.67)	0.116

The *p* values were obtained from the Chi-squared test

*The *p* values were obtained from the Mann–Whitney test

Bold values indicate statistically significant *p* values (*p* < 0.05)

Five patients who underwent LC died secondary to bile duct injury, postoperative bleeding, acute myocardial infarction, pulmonary embolism and septic shock due to cholecystitis. The two patients who died in the PCD group were from septic shock due to cholecystitis.

The sex, IMC, ASA Physical Status Classification, chronic obstructive pulmonary disease, cardiovascular disease, anticoagulant agents, antiplatelet agents, bile duct diameter and laparoscopic cholecystectomy were identified as potential risk factors for the development of major complications therefore, were included in the multivariate analysis. we did not find statistically significant differences in the outcomes with any of the variables (Table 3).

**Table 3:** Logistic regression for major complications (Clavien-Dindo ≥3)

	OR	CI95%	*p* value
Sex	1.949	0.455-8.340	0.368
Body mass index	1.004	0.838-1.204	0.963
ASA≥3	0.855	0.133-5.496	0.869
Chronic obstructive pulmonary disease	0.560	0.105-2.971	0.495
Cardiovascular disease	2.109	0.379-11.716	0.394
Anticoagulant agents	2.736	0.282-26.556	0.385
Antiplatelet agents	0.883	0.132-5.903	0.898
Bile duct diameter	0.957	0.818-1-120	0.585
Laparoscopic cholecystectomy	0.246	0.022-2.804	0.259

### Analysis of high-risk patients’ subgroup

Comparison of demographic, clinical and surgical variables in the subgroup of high-risk patients (defined as ASA ≥3) showed the same differences within the general group, except for gender (Table 4).

**Table 4:** Demographic, clinical, and surgical characteristics of high-risk patients (ASA ≥3) older than 90 years with cholecystitis, according to the procedure performed

	*N* (%) *N***=**71	Laparoscopic cholecystectomy (%) *n***=**44	Percutaneous catheter drainage (%) *n***=**27	*p* value
Age (median)(IQR)(years)	92 (4.00)	92 (3.00)	92 (4.00)	0.286*
Sex				0.100
Female	44 (61.97)	24 (54.55)	20 (74.07)	
Male	27 (38.03)	20 (45.45)	7 (25.93)	
Body mass index (median)(IQR)(kg/m^2^)	23.35 (5.45)	24.2 (5.80)	21.9 (3.50)	0.018*
Co-morbidity				
Diabetes mellitus	18 (25.35)	12 (27.27)	6 (22.22)	0.635
Arterial hypertension	57 (80.28)	36 (81.82)	21 (77.78)	0.678
Chronic obstructive pulmonary disease	33 (46.48)	20 (45.45)	13 (48.15)	0.825
Chronic kidney disease	13 (18.31)	7 (15.91)	6 (22.22)	0.504
Cardiovascular disease	31 (43.66)	12 (27.27)	19 (70.37)	**<0.001**
Liver disease	2 (2.82)	1 (2.27)	1 (3.70)	0.724
Charlson comorbidity index (median)(IQR)(points)	6.0 (2.00)	6.0 (2.00)	7.0 (3.00)	0.712*
Anticoagulants agents	8 (11.27)	2 (4.55)	6 (22.22)	**0.022**
Antiplatelet agents	26 (36.62)	11 (25.00)	15 (55.56)	**0.009**
Pre-operative laboratories (median)(IQR)				
Leukocytes (x10^3^)	13.30 (6.20)	13.00 (5.72)	13.62 (10.88)	0.183*
Hemoglobin (mg/dL)	13.3 (2.60)	13.5 (2.85)	13.1 (2.60)	0.598*
Bilirubins (mg/dL)	1.12 (1.35)	1.06 (0.97)	1.29 (1.62)	0.368*
Alkaline phosphatase (mg/dL)	121 (92.00)	135.5 (102)	111 (98.00)	0.808*
Aspartate aminotransferase (mg/dL)	27 (55.00)	27.5 (56.75)	26.0 (56.00)	0.981*
Alanine aminotransferase (mg/dL)	31 (67.00)	30.5 (86.50)	34.0 (50.00)	0.557*
Bile duct diameter (median)(IQR)(mm)	10.35 (7.72)	13 (5.72)	6.45 (3.00)	<0.001*
Classification of severity of cholecystitis				0.015*
I	4 (5.63)	4 (9.09)	0 (0.00)	
II	21 (29.58)	17 (38.64)	4 (14.81)	
III	46 (64.79)	23 (52.27)	23 (85.19)	
Pre-operative ERCP				0.424
No	60 (84.51)	36 (81.82)	24 (88.89)	
Yes	11 (15.49)	8 (18.18)	3 (11.11)	
Time from admission to surgical procedure (median)(IQR) (days)	5.0 (4.00)	5.0 (4.00)	4.0 (5.00)	0.578

The *p* values were obtained from the Chi-squared test

*The *p* values were obtained from the Mann–Whitney test

Bold values indicate statistically significant *p* values (*p* < 0.05)

There were also no statistically significant differences between outcomes for both treatment groups in this subgroup, with a complication rate of 43.18%, a major complication rate of 22.73%, and a mortality rate of 4.55% for patients who underwent LC (Table 5).

**Table 5:** Surgical outcomes in high-risk patients (ASA ≥3) according to the procedure performed

	*N* (%) *N***=**71	Laparoscopic cholecystectomy (%) *n***=**44	Percutaneous catheter drainage (%) *n***=**27	*p* value
Conversion from laparoscopic to open				
No	37 (52.11)	37 (84.09)	Not apply	
Yes	7 (9.85)	7 (15.91)		
Type of cholecystectomy				
Total	34 (47.88)	34 (77.27)	Not apply	
Subtotal	10 (14.08)	10 (22.73)		
Drain use				
No	31 (43.66)	31 (70.45)	Not apply	
Yes	13 (18.30)	13 (29.55)		
Hospital stay (median)(IQR)(days)	10.0 (7.00)	10.5 (7.00)	10.0 (6.00)	0.614
Need for reintervention				0.260
No	65 (91.55)	39 (88.64)	26 (96.30)	
Yes	6 (8.45)	5(11.36)	1 (3.70)	
Clavien-Dindo				0.548
I	8 (11.27)	6 (13.64)	2 (7.41)	
II	5 (7.04)	3 (6.82)	2 (7.41)	
IIIA	4 (5.63)	3 (6.82)	1 (3.7)	
IIIB	3 (4.23)	3 (6.82)	0 (0.00)	
IV	2 (2.82)	2 (4.55)	0 (0.00)	
V	4 (5.63)	2 (4.55)	2 (7.41)	
Any complication	26 (36.62)	19 (43.18)	7 (25.93)	0.143
Major complications (Clavien-Dindo ≥3)	13 (18.31)	10 (22.73)	3 (11.11)	0.219

The *p* values were obtained from the Chi-squared test

*The *p* values were obtained from the Mann–Whitney test

Bold values indicate statistically significant *p* values (*p* < 0.05)

## Discussion

Our study did not show statistically significant differences when comparing outcomes in LC and PCD for the management of cholecystitis in a series of 96 super elderly patients. Neither were there any statistically significant differences when analyzing a subgroup of high-risk patients within the series. We consider the results of this paper as a contribution to the available literature regarding treatment alternatives for patients over 90 years of age, regarding insufficient evidence in this group of patients when choosing adequate treatment between both procedures (LC or PCD).

LC is considered the standard of care for benign biliary disease due to its efficacy and low complication rate, however we should regard special considerations for this procedure in elderly patients because of the high complication and mortality rate in this age group [2]. Furthermore, the rate of difficult cholecystectomies in this age group according to Nassar’s Score were higher than what is usually reported [8, 14].

For high-risk patients, PCD has been proposed as an alternative procedure since it can be performed at the patient’s bedside and under local anesthesia, with the aim of reducing risks of general anesthesia and those inherent to the surgical procedure [15, 16]. However, studies have shown a higher rate of complications in PCD for the management of acute cholecystitis compared to LC [17], even in elderly and high-risk patients, therefore, some guidelines recommend LC as the standard of care [2]. The current evidence-based indication for PCD is in patients not eligible for surgical procedure in order to resolve septic status [1].

When evaluating the subgroup of high-risk patients (ASA≥3) we found surgical outcomes similar to those observed in the general group, with no statistically significant differences between the two procedures. It should be noted that, when assessing the high-risk subgroup, only 3 patients (11.11%) of those who underwent PCD were excluded from the analysis; on the other hand, 21 patients (30.30%) of those who underwent LC were; this suggests that PCD is used in patients who are considered unable to tolerate surgery, which is logical and consistent with actual evidence.

We also highlight our finding of a statistically significant difference in patients with a greater presence of cardiovascular and pulmonary comorbidities who underwent PCD. We believe that this difference showcases both surgeon and anesthesiologist preference for PCD in these patients during clinical practice, taking into consideration the physiological repercussions of pneumoperitoneum during laparoscopic procedures.

Regarding the limitations of this paper, we consider the retrospective nature of the cohort, small sample size and limited follow-up. The deficient information on the medium- and long-term follow-up of patients in the group who underwent PCD is particularly relevant, since the monitoring of complications such as catheter dysfunction/removal or symptom recurrence that may deserve additional interventions in the medium and long term is essential for a complete and accurate description of results in this group.

The super elderly group constitutes a special population, which is constantly increasing (1,5)(1,5)(1,5)(1,3), and deserves specialized studies due to their characteristics [18, 19]. Benign biliary pathology is not the only exception and complications associated to cholelithiasis and age should be taken into account [20]. To this date, LC continues to be the standard of care for benign biliary pathology [20]; however, prospective studies with longer follow-up controls are needed in this group of patients, considering LC may not be so well tolerated, and to evaluate factors such as quality of life [21] with the aim of defining the best treatment alternative.

## Conclusions

In this study, we found no significant differences between major complications and mortality in super elderly patients with acute cholecystitis treated by LC or PCD. Although LC is still the standard of care in benign biliary pathology, morbidity and mortality rates associated with this procedure increase exponentially with age, therefore, it is necessary to evaluate each case individually to select the best treatment alternative available in order to avoid complications and improve quality of life in super elderly patients.

## Data Availability

Data are available on request through institutional review board of Hospital Universitario Mayor - Méderi. You can contact to request the data to jose.daza@mederi.com.co.
